# Variants in the *PRPF8* Gene are Associated with Glaucoma

**DOI:** 10.1007/s12035-017-0673-5

**Published:** 2017-07-13

**Authors:** Shazia Micheal, Barend F. Hogewind, Muhammad Imran Khan, Sorath Noorani Siddiqui, Saemah Nuzhat Zafar, Farah Akhtar, Raheel Qamar, Carel B. Hoyng, Anneke I. den Hollander

**Affiliations:** 1 Department of Ophthalmology, Donders Institute for Brain, Cognition and Behaviour, Radboud University Medical Center, P.O. Box 9101, 6500 HB Nijmegen, The Netherlands; 2 Department of Clinical Genetics, Academic Medical Centre, Amsterdam, the Netherlands; 3 Department of Human Genetics, Donders Institute for Brain, Cognition and Behaviour, Radboud University Medical Center, Nijmegen, the Netherlands; 4 Department of Pediatric Ophthalmology, Al-Shifa Eye Trust Hospital, Jhelum Road, Rawalpindi, Pakistan; 5 Department of Biosciences, COMSATS Institute of Information Technology, Islamabad, Pakistan; 6 Department of Biochemistry, Al-Nafees Medical College & Hospital, Isra University, Islamabad, Pakistan

**Keywords:** Primary open angle glaucoma, Whole exome sequencing, Variant, Pathogenic, PRPF8

## Abstract

Glaucoma is the cause of irreversible blindness worldwide. Mutations in six genes have been associated with juvenile- and adult-onset familial primary open angle glaucoma (POAG) prior to this report but they explain only a small proportion of the genetic load. The aim of the study is to identify the novel genetic cause of the POAG in the families with adult-onset glaucoma. Whole exome sequencing (WES) was performed on DNA of two affected individuals, and predicted pathogenic variants were evaluated for segregation in four affected and three unaffected Dutch family members by Sanger sequencing. We identified a pathogenic variant (p.Val956Gly) in the *PRPF8* gene, which segregates with the disease in Dutch family. Targeted Sanger sequencing of *PRPF8* in a panel of 40 POAG families (18 Pakistani and 22 Dutch) revealed two additional nonsynonymous variants (p.Pro13Leu and p.Met25Thr), which segregate with the disease in two other Pakistani families. Both variants were then analyzed in a case-control cohort consisting of Pakistani 320 POAG cases and 250 matched controls. The p.Pro13Leu and p.Met25Thr variants were identified in 14 and 20 cases, respectively, while they were not detected in controls (*p* values 0.0004 and 0.0001, respectively). Previously, *PRPF8* mutations have been associated with autosomal dominant retinitis pigmentosa (RP). The *PRPF8* variants associated with POAG are located at the N-terminus, while all RP-associated mutations cluster at the C-terminus, dictating a clear genotype-phenotype correlation.

## Introduction

Glaucoma is an irreversible optic neuropathy characterized by progressive degeneration of retinal ganglion cells (RGCs). It affects more than 70 million people worldwide with approximately 10% being bilaterally blind [1]. Glaucoma is also called a silent thief of the sight due to the damage of the peripheral vision first. Since glaucoma is typically asymptomatic until a substantial loss of vision has occurred, an even higher number of people is affected than the numbers estimated worldwide [2, 3]. Typically, glaucoma is classified as primary open angle (POAG) and angle closure glaucoma (PACG). POAG is the most common type of glaucoma affecting about 1–2% of individuals over the age of 40, with a higher prevalence among African individuals [4–6].

Despite the fact that glaucoma has different types and distinct etiologies, the death of the RGCs is a unifying theme, together with visual field defects and a characteristic optic nerve excavative atrophy [7, 8]. Since many years, research efforts have been made to elucidate the molecular mechanisms of the progressive optic nerve degeneration, but the underlying causes of the disease still remain poorly understood. Genome-wide association studies in case-control cohorts have identified several genetic variants associated with POAG, but they explain only a small proportion of the genetic load [9]. Although more than 15 loci have been identified for glaucoma till date, only five genes have been identified with the causative mutations which include the following: *MYOC*/*TIGR* [10, 11], *OPTN* [12, 13], *ASB10* [14, 15], *WDR36* [16], and *EFEMP1* [17]. Mutations in sixth gene *CYP1B1* were initially identified in the patients with the primary congenital glaucoma but later association has been reported in sporadic cases and families with both juvenile and adult-onset POAG. Mutations in *MYOC* are responsible for disease only in 4% of the JOAG and POAG cases with raised intraocular pressure (IOP) in an autosomal dominant mode of inheritance [11, 18]. The overall prevalence of *OPTN* mutations in POAG is 0.4%, and the role of *WDR36* is still contradictory in glaucoma, even no difference in the phenotype was observed between the wild type and heterozygous mice for the *WDR36* which makes it a weaker candidate for glaucoma. However, *ASB10* [14, 15] and *EFEMP1* [18, 19] were recently identified and the prevalence of patients with mutations in these genes is difficult to conclude. Overall, it has been estimated that less than 10% of POAG cases have pathogenic mutations in one of these genes. This suggests that a substantial percentage of patients may carry mutations in genes yet to be identified [20].

Following this rationale, we performed whole exome sequencing (WES) in two affected individuals of a family with adult-onset POAG to find the causative gene for this family.

## Materials and Methods

### Subjects

Patients were recruited at the glaucoma departments of Radboud University Medical Center, The Netherlands and Al-Shifa Eye Trust Hospital, Pakistan. The study was approved by the Institutional Review Boards of the Department of Ophthalmology, Radboud University Medical Center and Al-Shifa Eye Trust Hospital, and adhered to the tenets of the Declaration of Helsinki. The families included in the study have at least two affected individuals in the family. The sporadic POAG patients were included based on the absence of any incidence of glaucoma among the relatives of the patient. Written informed consent was obtained from affected and unaffected participants and/or their parents to participate in the study and for blood withdrawal. Genomic DNA was extracted using AutoPure LS DNA Extractor and PUREGEN reagents (Gentra Systems Inc., Minneapolis, MN, USA).

### Clinical Examination

Complete ophthalmic examinations were performed for both sporadic and familial patients. The diagnosis of the POAG was made when the following criteria were met: briefly, absence of secondary glaucoma, an open anterior chamber angle by gonioscopy (Shaffer grade III or IV), higher IOP (>22 mmHg) measured using Goldmann applanation tonometry, a cup-to-disc ratio (CDR) >0.7 with thinning or notching of the disc rim, and nerve fiber layer defects. Visual field defects typical of glaucoma were determined with a Humphrey Field Analyzer (Zeiss Humphrey Systems, Dublin, CA, USA) and includes arcuate scotoma, nasal step, paracentral scotoma, and generalized depression. Only individuals affected with advanced primary open angle glaucoma were included in the study while normal tension glaucoma patients were excluded. The controls included in the study also underwent the clinical examination, and only individuals with the normal vision without any eye anomaly and no family history of glaucoma were included in the study.

### Whole Exome and Sanger Sequencing

Whole exome sequencing (WES) was performed in two affected individuals of family A, (II:3 and II:4, Fig. 1a) with adult-onset POAG. The study adhered to the principles of the declaration of Helsinki. Written informed consent was obtained prior to the study. Genomic DNA was extracted from the peripheral leukocytes of the family members. Enrichment of exonic sequences was achieved by using the SureSelectXT Human All Exon V.2 Kit (50 Mb), (Agilent Technologies, Inc., Santa Clara, CA, USA). Sequencing was performed on a SOLiD 4 sequencing platform (Life Technologies, Carlsbad, CA, USA). The hg19 reference genome was aligned with the reads obtained using SOLiD LifeScope software V.2.1 (Life Technologies). The identified variants were validated and segregation analysis was performed in all available family members using standard PCR and Sanger sequencing. Sequencing was performed using the Big Dye Terminator Cycle Sequencing-Ready Reaction Kit (Applied Biosystems) on a 3730 DNA automated sequencer (Applied Biosystems, Foster City, CA, USA) using standard protocols.

**Fig. 1:**
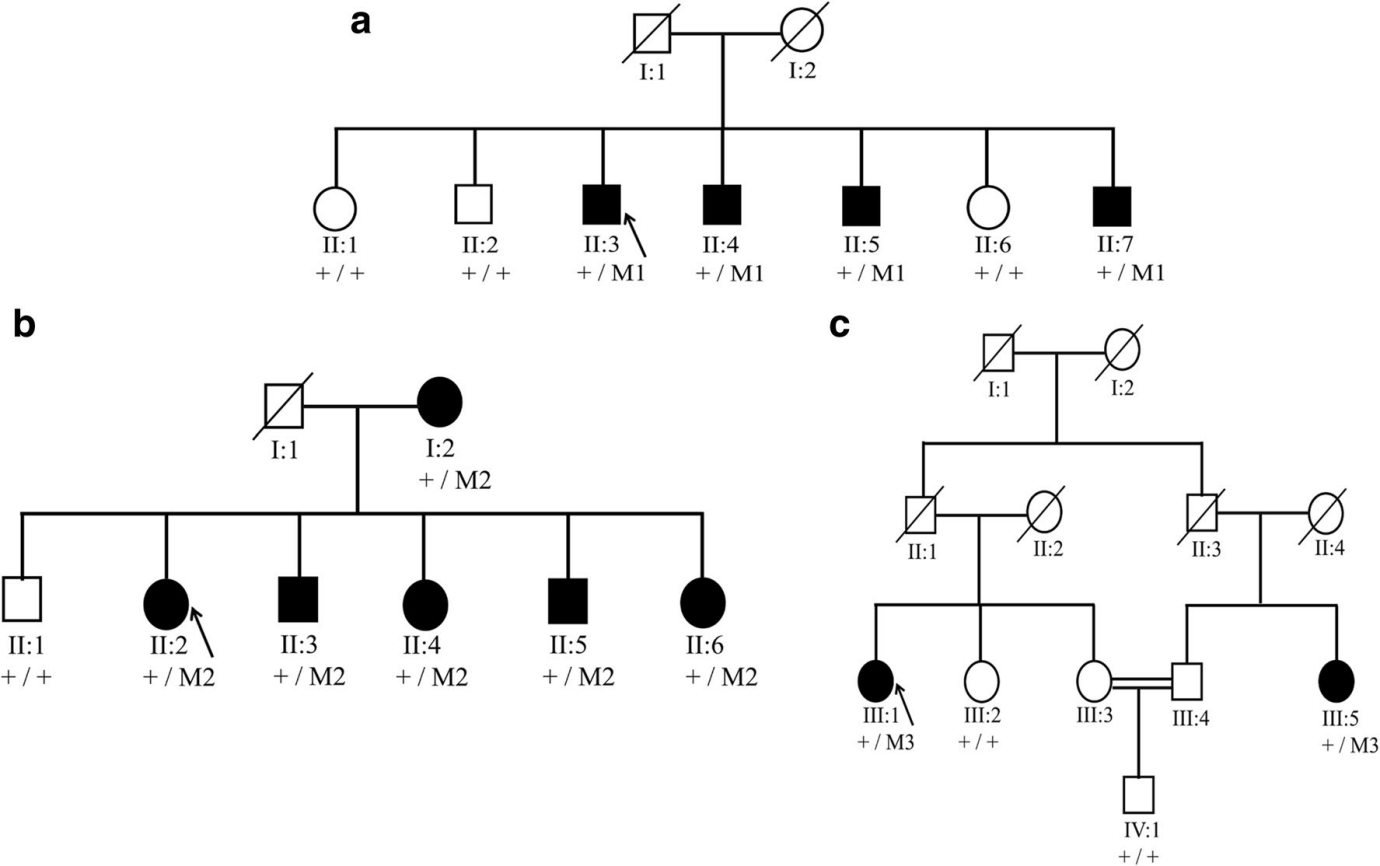
Segregation of *PRPF8* variants in glaucoma families. **a** Family A with the heterozygous mutation c.2894T>G; p.Val965Gly (M1) in the *PRFP8* gene. **b** Family B showing segregation of the variant (c.38C>T; p.Pro13Leu (M2). **c** Family C with the heterozygous variant c.74T>C; p.Met25Thr (M3) segregating with the disease

### Data Processing

To identify the causative variant in this family, only variants shared by both affected individuals were considered for further analysis. All variants present within intergenic, intronic, and untranslated regions and synonymous substitutions were excluded. In addition, variants with an allele frequency >0.5 in public databases, including the dbSNP132 (http://www.ncbi.nlm.nih.gov/projects/SNP/) and Exome Aggregation Consortium (ExAC http://exac.broadinstitute.org/) databases, were excluded. Variants that were predicted not to be pathogenic by in-silico prediction including Sorting Intolerant from Tolerant (SIFT http://sift.bii.a-star.edu.sg/), MutationTaster (http://www.mutationtaster.org/), and Polymorphism Phenotyping (PolyPhen-2 http://genetics.bwh.harvard.edu/pph2/), variants with a low PhyloP score (<2.7) or a low Grantham score (<80) were also excluded. The variants that remained after these filtering steps were validated by Sanger sequencing, and segregation analysis was performed in the family. Amino acid conservation of mutated residue among the orthologous species was assessed by performing the aligned using Vector NTI Advance (TM) 2011 software. The amino acid sequences were obtained from protein sequence database UniProt (http://www.rcsb.org/pdb/protein/Q6P2Q9).

## Results

### Mutation Identification

**Fig. 2:**
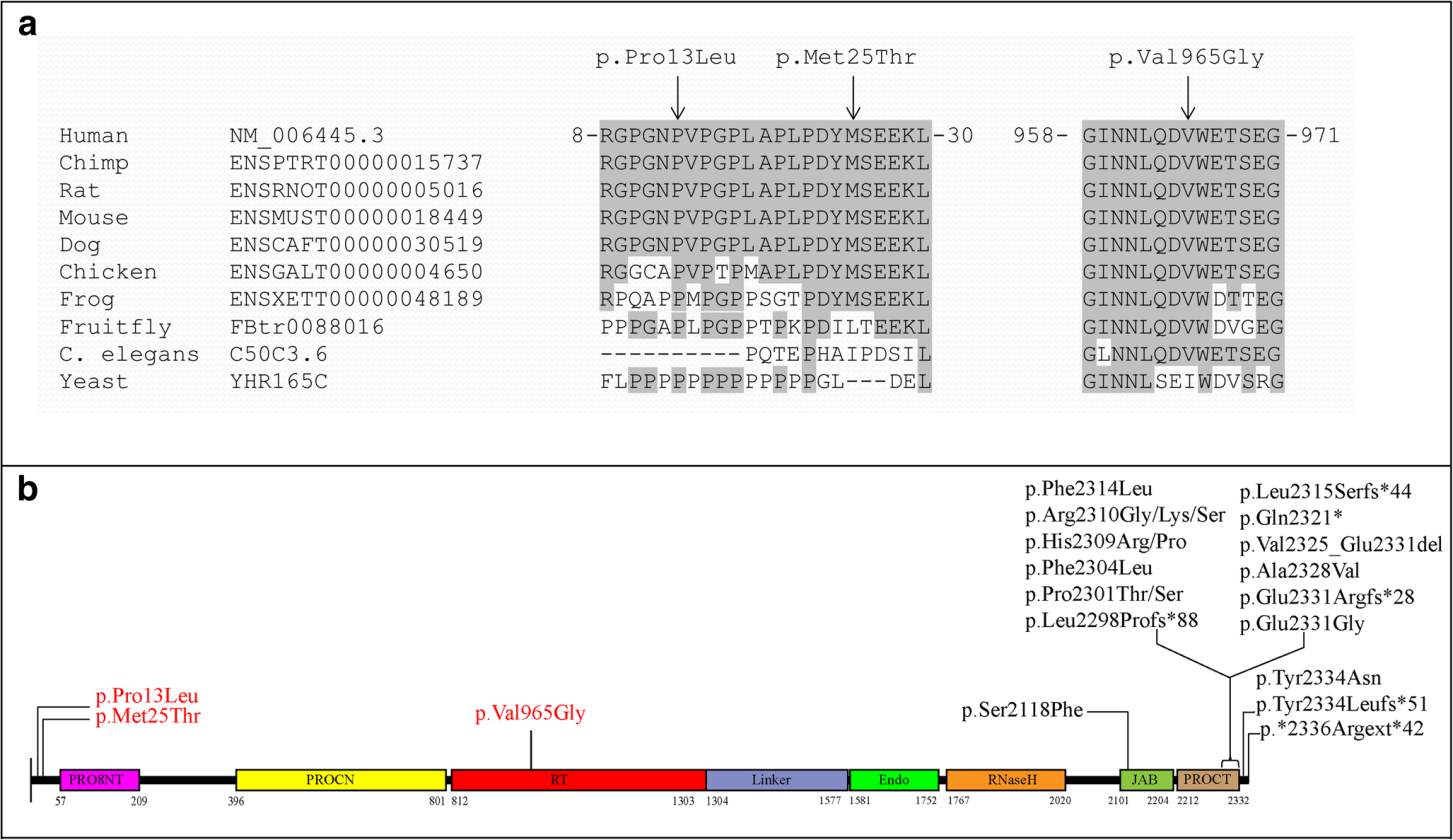
**a** Multiple sequence alignment of PRPF8 orthologues. Conserved amino acids are shaded, and the positions of mutated amino acids p.Pro13Leu (P), p.Met25Thr (M), and p.Val965Gly (V) are indicated with an *arrow*. **b** Distribution of PRPF8 mutations in different domains. Conserved domains, PrP8 N-terminal domain (PRO8NT), central N-terminal domain in pre-mRNA splicing factors of PRO8 family (PROCN), reverse transcriptase homology domain (RT), restriction endonuclease homology domain (Endo), ribonuclease H homology domain (RNase H), JAB1/Mov34/MPN/PAD-1 ubiquitin protease (JAB), C-terminal domain in pre-mRNA splicing factors of PRO8 family (PROCT). Mutations (indicated in *red*) associated with autosomal dominant (ad) POAG are all located at the C-terminus of the protein while adRP mutations (indicated in *black*) are all located at the N-terminus

Only the rare variants shared between two affected individuals of the family were further considered for validation by Sanger sequencing (Table 1). Segregation analysis was performed for >20 variants based on the *in-silico* prediction and expression in the eye. Only one novel variant (c.2894T>G; p.Val965Gly) in the *PRPF8* gene was identified that segregates with the disease in family A (Fig. 1a). This variant was predicted to be deleterious by SIFT, probably damaging by PolyPhen-2 and disease causing by Mutation Taster. The wildtype nucleotide was highly conserved (phyloP score 5.13), and the amino acid residue p.Val965 was highly conserved among different orthologues (Fig. 2a). The p.Val965Gly variant was not present in the dbSNP132 or ExAc databases, nor was it identified in 150 Dutch control individuals.

**Table 1:** Number of variants identified by WES in two affected individuals of family A

Filtering Steps	Individual II:3	Individual II:4	Shared variants for both individuals
Total variants	45.953	46.860	31.622
SNP frequency <0.5	29.020	29.688	16.645
In-house frequency <0.5	2.431	2.519	476
Exonic and canonical splice sites	887	900	175
Nonsynonmous	646	638	123
Grantham score >80	281	274	41
Phylop >2.7	234	231	46

### Clinical Findings of Family A

**Fig. 3:**
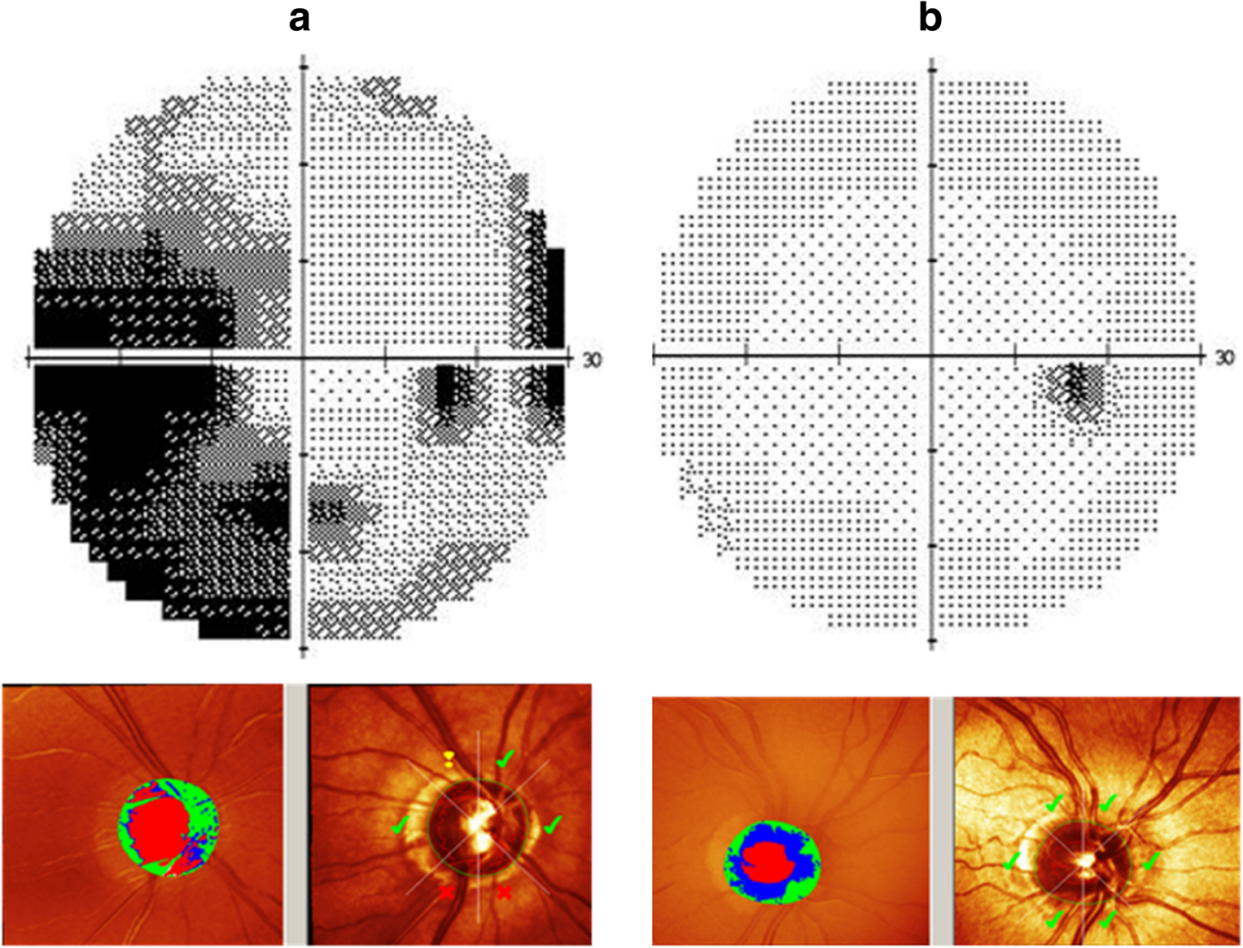
Phenotypic characterization of the right eyes of two representative individuals of family A with the pathogenic variant in the *PRPF8* gene. Each panel has two parts; the upper part depicts the visual field printouts of the Humphrey Field Analyzer (HFA), and the lower part shows screenshots from Heidelberg Retina Tomograph II (HRT) analysis to detect loss of the papillary neuroretinal rim. **a** HFA and HRT results for the 67-year-old proband (individual II-3 of family A) are shown. The large dark areas in the HFA results correspond to glaucomatous scotomas due to nerve fiber layer defects. HRT scans demonstrate glaucomatous increased optic disc cupping and suspect (*exclamation marks*) or manifest pathological (*crosses*) neuroretinal rim measures (according to the Moorfield’s regression analysis) within the different quadrants of the optic disc. **b** HFA and HRT results for a 61-year-old, unaffected sibling (individual II-2 of family A) of the proband are shown for comparison. HFA results indicate that there is no darkening due to glaucomatous defects on HFA. The small temporal black area corresponds to the physiological blind spot. HRT results (with only *green tick marks*) show no thinning of the neuroretinal rim

The four affected individuals of family A (Fig. 1a) were diagnosed with POAG. They all had bilateral glaucomatous optic neuropathy with a cup-to-disc ratio (CDR) >0.7 on fundoscopy with compatible glaucomatous visual field loss. The intraocular pressure (IOP) was >22 mmHg, and the anterior chamber angles were open in all affected individuals. They also showed abnormal results on Heidelberg Retina Tomography (HRT) II testing. The visual field and the HRT analysis of the 67-year-old proband (family A, individual II:3) and those of a 60-year-old, unaffected male sibling (family A, individual II:2) are shown in Fig. 3. The three unaffected siblings did not show any (glaucomatous) optic neuropathy nor visual field loss as present in the four affected siblings.

### Panel Screening for PRPF8

Sanger sequencing of the entire open reading frame of *PRPF8* in a cohort of 40 adult-onset POAG families (*n* = 18 Pakistani and *n* = 22 Dutch) having at least two affected individuals was performed. Sequencing identified two additional nonsynonymous variants, c.38C>T; p.Pro13Leu and c.74T>C; p.Met25Thr, which segregate with the disease in families B and C, respectively (Fig. 1b, c). Affected individuals in both families were diagnosed with adult-onset POAG, with an IOP >21 mmHg and a CDR >0.7.

Both variants are localized in exon 2 of the *PRPF8* gene. Therefore, exon 2 was Sanger sequenced in a case-control cohort consisting of 320 Pakistani POAG patients and 250 Pakistani controls. The p.Pro13Leu and p.Met25Thr variants were identified in 14 and 20 cases, respectively, while they were not detected in controls (*p* values 0.0004 and 0.0001, respectively). The p.Pro13Leu and p.Met25Thr variants were present in the ExAC database, with allele frequencies of 0.00014 (16/113928 individuals) for p.Pro13Leu and 0.00002 (3/115076 individuals) for p.Met25Thr, respectively. The wildtype nucleotide and amino acid residues are highly conserved among different orthologues (Fig. 2a).

## Discussion

The precursor mRNA-processing factor 8 (PRPF8) is the core component of the U5 snRNP. It is the largest and most evolutionarily conserved protein, central to the dynamics of the spliceosome [21, 22]. As a key part of the catalytic core of the spliceosome, it not only makes direct interactions with the 5′ splice site, branch point, and 3′ splice site in the pre-mRNA, but also engages the U5 and U6 snRNAs and the excised intron [23, 24]. Previous studies have indicated a crucial role of PRPF8 in the vast majority of pre-mRNA splicing and its requirement in all tissues [25, 26]. PRPF8 is responsible for processing of the majority of intron-containing transcripts, including alternatively spliced mRNAs in higher eukaryotes [27]. Mutations in human *PRPF8* that affects spliceosome assembly and function are found in autosomal dominant retinitis pigmentosa (RP) (OMIM 600059), characterized by a progressive degeneration of the rod and cone photoreceptors in the retina [28–30]. All germline mutations reported in the *PRPF8* gene in patients with RP are clustered at the C-terminus of the protein.

PRPF8 interacts with other proteins at both the N-terminal and C-terminal of the protein. Mutations previously identified in RP are all localized at the C-terminus of the protein and affect the binding of the interacting partners with the C-terminus of the PRPF8 protein. Pathogenic mutations in the Jab1/MPN domain of human PRPF8 have been described in RP [28], and mutations of equivalent residues in the yeast Jab1/MPN domain disrupt its interaction with Brr2 [31]. Brr2 is involved in catalyzing the separation of the U4/U6 snRNA duplex [32]. In addition, the *prp8–1* allele G2347D was observed to have a detrimental effect on the interaction of Prp8p with Brr2 in yeast two-hybrid and coimmunoprecipitation assays [33]. These studies help to elucidate that mutations involved in RP at the C-terminus of the PRPF8 disrupt the interactions with the interacting partners important for the splicing.

In the current study, we identified mutations located at the N-terminus of PRPF8 associated with glaucoma. We postulate that these variants can disrupt the interaction of PRPF8 with its interacting partners at the N-terminus of the protein, such as PRP39 and PRP40 [22]. All three variants identified in glaucoma are predicted to be pathogenic using different pathogenicity programs and reside within these interacting domains. Biochemical studies are needed to determine whether these variants indeed interrupt these interactions.

Human mRNA expression studies have shown that *PRPF8* is highly expressed in the retinal inner nuclear layer containing the bipolar cells, horizontal cells, amacrine cells, and Müller glia cells, as well as in the retinal ganglion cell layer. In the photoreceptor cells, the expression of *PRFP8* is comparatively lower [34]. POAG is characterized by loss of retinal ganglion cells (RGCs), large and complex cells extending from the inner retina. The convergence of the axons of RGCs at the optic disc creates the neuroretinal rim. In POAG, the loss of the RGC axons leads to progressive thinning of this neuroretinal rim of the optic nerve, thereby enlarging the optic nerve cup. Since *PRPF8* is highly expressed in RGC axons, pathogenic variants in *PRPF8* could affect the function of the spliceosomal machinery in these cells and thus induce POAG.

The identification of three variants in *PRPF8* suggests that POAG may be a splicing disease. The *PRPF8* variants associated with POAG are located at the N-terminus, while all RP-associated mutations cluster at the C-terminus, dictating a clear genotype-phenotype correlation.
